# Simulation of Monopulse Radar Under Jamming Environments Based on Space Slicing

**DOI:** 10.3390/s25185785

**Published:** 2025-09-17

**Authors:** Shaoning Lu, Yuefeng Deng, Liehu Wu, Qile Li, Guodong Qin

**Affiliations:** 1 College of Computer Science and Technology, Nanjing University of Aeronautics and Astronautics, Nanjing 211106, China; lushaoning2013@163.com; 2 School of Electronic Engineering, Xidian University, Xi’an 710071, China; 23021211777@stu.xidian.edu.cn (Y.D.); wuliehu560@163.com (L.W.); liqilecx@yeah.net (Q.L.)

**Keywords:** monopulse radar, suppression jamming, deceptive jamming, space slicing, radar simulation

## Abstract

Under jamming environments, the simulation system of monopulse radar consumes substantial computational resources due to echo signal and jamming signal generation, as well as real-time radar signal processing, leading to large time consumption in evaluating the radar’s anti-jamming performance in complex electromagnetic jamming scenarios. This paper proposes a monopulse radar simulation strategy based on space slicing to improve simulation efficiency. By considering the operational characteristics of the monopulse radar, including search, acquisition, track, and narrow search modes, the space where radar and target are located is divided into discrete grid points in different granularity. The simulation results for each slice are used to replace full-process real-time signal processing, thus improving the overall efficiency of the simulation system. The estimation errors of the target’s range, velocity, and angular after space slicing are theoretically analyzed. Simulation experiments demonstrate that, by utilizing the proposed space slicing strategy, the simulation speed is improved dramatically, with target parameter estimation errors remaining relatively small compared to full-process real-time simulations.

## 1. Introduction

Monopulse radar enables the measurement of target range, azimuth, and elevation angles through the transmission of a single pulse. This technique is characterized by its simplicity, high real-time performance, and robust anti-jamming capabilities, making it widely applicable in fields such as guidance, navigation, and target detection [1,2,3,4,5].

With the increasing complexity of the electromagnetic environment, radar echo signals often include significant jamming, such as suppression jamming [6,7] and deceptive jamming [8,9,10,11,12,13]. Although the jamming signals are weakened by properly allocating the radar resources [14,15,16], the remainder jamming signals present substantial challenges to the radar target detection. Many anti-jamming methods in the monopulse radar are proposed to improve the target detection performance. A spatial filter for main-lobe jamming cancellation is designed in [6] to cancel the main-lobe jamming while keeping the target signal power unchanged. A novel distributed array nodes configuration is designed to address the distortion on the monopulse curve resulting from adaptive beamforming in the presence of mainlobe and sidelobe interferences [7]. A Doppler compensation method is used to construct the range–Doppler spectrum of the monopulse echo, in which the true and false targets can be effectively identified [8]. By using the difference between sum and difference beam pattern in mono-pulse radar, the polarization decomposition and polarization estimation method of single polarization returns are studied in [9]. The interference in sum and difference channel is suppressed. Although many anti-jamming research fruits appear, these algorithms mostly only suppress interferences under some specific scenarios.

To thoroughly evaluate the radar anti-jamming performance, a radar simulation system is usually constructed under the different jamming environment. A detailed modeling and simulation of the air-to-ground ranging performance of airborne monopulse radar system over complex terrain is proposed in [17]. In this paper, time-domain monopulse signals reflected by the terrain’s surface are modeled using the dynamics of the airborne platform, clutter properties, and radio frequency specifications of the radar. In [18], MATLAB, Simulink, and RF Blockset are used to build a model for a monopulse tracking system with the integration of transmitter antenna, receiver antenna, and comparator. The co-simulation method of Systems Tool Kit (STK) and MATLAB is proposed to simulate the detection range of enemy radar suppressed by the jammer in the three-dimensional electronic warfare environment in [19]. In [20], unmodulated pulse radar and four types of communication interference signal are designed to show the possibility of radar interference effect analysis using simulation.

Despite the relatively simple signal processing flow of monopulse radar, the short radar pulse repetition interval (PRI) and high sampling rate result in significant time consumption in the simulation system. Especially, this time consumption will be aggravated dramatically in the anti-interference evaluation based on the radar system simulation under all kinds of jamming scenarios. Therefore, enhancing the simulation efficiency of monopulse radar systems under the jamming scenarios is one of the key challenges in the field of radar simulation today.

High-performance real-time processing hardware circuit boards can improve the computational efficiency of monopulse radar simulation systems [21,22]. For instance, the Digital Signal Processor (DSP) and Field Programmable Gate Array (FPGA) architecture has been applied to monopulse radar signal processing to improve computational efficiency [21], while a parameterized and software-reconfigurable DSP and FPGA high-performance real-time processing board has been employed for dual-PRI monopulse radar signal processing [22]. However, this approach is costly and lacks flexibility and scalability. An alternative approach involves the parallel computing capabilities of processors, such as central processing units (CPUs) and Graphics Processing Units (GPUs). GPUs, due to their parallel thread execution and synchronization techniques [23,24], have proven effective in accelerating programs through parallel computation [25,26,27,28]. A short-track real-time imaging scheme with GPU architecture and processing method is proposed in [23]. In [27], a parallel simulation system for the MIMO radar based on the CPU/GPU architecture is designed to accelerate the signal processing flow. Nevertheless, the efficiency of parallel computing remains constrained by the number of processors and the design of parallel computing algorithms.

The aforementioned methods primarily focus on improving simulation efficiency through enhanced chip computational capabilities. This paper, however, proposes a novel approach by investigating the operation processes of monopulse radar and innovating the simulation algorithm to improve system efficiency. The space where radar and target locate is divided into discrete slices at a certain granularity for each radar operation state. The middle part upon multiple estimations is chosen as the representation to decrease the data rate, which improves the computational efficiency. It is important to note that the signal parameters and resolution vary across different radar operational states, necessitating distinct slicing strategies for each.

The main contributions of this paper are summarized as follows:

(1) A novel space slicing approach is proposed to improve the computational efficiency of the monopulse radar simulation system under the jamming scenarios. The slicing strategies for the search, acquisition, track, and narrow state are provided. The slicing granularities for each operating state are theoretically analyzed. In the track state, the center of the slice is used to represent the slice to decrease the data rate. Once the slice data of each state under different jamming types is generated, the monopulse radar simulation based on the slice data selection algorithm is performed to acquire the target’s range, velocity, and angular information quickly according to different demands.
(2) The expressions of the target’s range, velocity, and angular error with spacing slicing method in the different states is given and discussed. In the track state, the range error is produced since the center of the slice is represented. The angular and velocity error is relatively small because the angular and velocity variation is very small in a slice, which leads to high precision with the proposed method.
(3) The simulations of the proposed method under the suppression and deceptive jamming, including broadband blocking jamming, range–velocity gate pull off (R-VGPO) jamming, and angular deceptive jamming, are provided in this paper.

The rest of this paper is structured as follows. Section 2 briefly introduces the signal processing of monopulse radar, including the models of jammer signals. Section 3 primarily proposes the space slicing method for each radar state and analyzes the theoretical errors of target parameter estimation of the proposed method. Section 4 evaluates the performance of the proposed method through numerical simulations. Section 5 concludes the paper.

## 2. Signal Processing Flow of Monopulse Radar in Jamming Environments

### 2.1. Signal Processing Flow of Monopulse Radar

Radar echo signals consist of both useful target signals and unwanted signals such as clutter, jamming, and noise. These unwanted signals can directly affect the radar’s operation and degrade its target detection performance. Therefore, a series of radar processing algorithms are required to enhance the signal-to-noise ratio (SNR), mitigate jamming from unwanted signals, and improve target detection capabilities.

**Figure 1:**
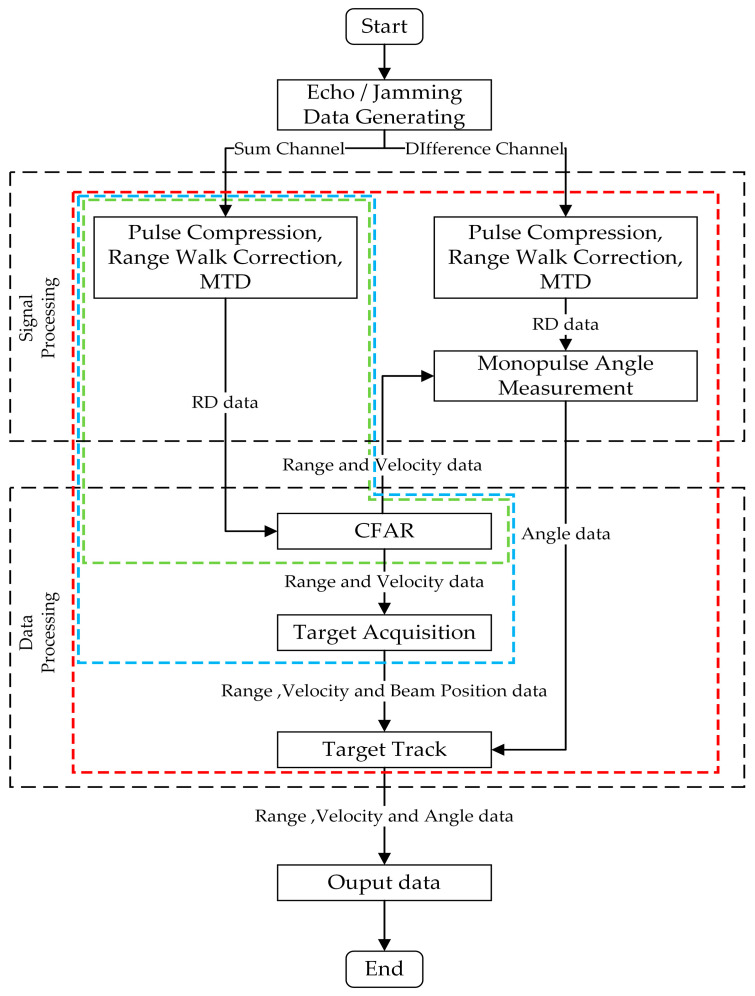
Signal processing flowchart of monopulse radar.

As illustrated in Figure 1, the signal processing flow of monopulse radar includes signal processing and data processing [18,21,22]. After producing the echo and jamming signal data, the sum and difference channel data modulated by antenna pattern are used to perform signal process, including pulse compression, range walk correction, moving target detection (MTD), and monopulse angle measurement. Then, the range–Doppler (RD) data is obtained and used to perform data processing, including constant false alarm rate (CFAR) detection, target acquisition, and track. Finally, the range, velocity, and angular data are obtained and outputted. The processing process in Figure 1 is the classical method in the monopulse radar, which is called full-process method in this paper [29,30].

It is noted that radar systems can generally be categorized into search state, acquisition state, track state, and narrow search state. The narrow search state operates similarly to the search state, except the operating parameters, which are ignored in Figure 1. In Figure 1, the green, blue, and red boxes represent the signal processing and data processing workflows for the search state, acquisition state, and track state, respectively.

#### 2.1.1. Signal Processing

This subsection comprises pulse compression, range walk correction, MTD, and monopulse angle measurement. The primary purpose of these steps is to improve the SNR of the echo signals, enhance target detection capabilities, and provide preliminary target information. The radar receiver features sum and difference channels (azimuth and elevation difference channels), with both channels independently performing the aforementioned processing steps. The information from the sum and difference channels is then combined during monopulse angle measurement to determine the target’s angular.

Pulse compression applies matched filtering at the receiver to compress the echo signal’s time width, thereby improving range resolution. Range walk correction compensates for target motion through the range resolution cell using frequency-domain correction methods to restore the target’s accurate position. MTD processes the target echo signals coherently across multiple pulses, accumulating energy over multiple pulses to enhance the signal strength of weak targets [31]. This is typically achieved through Doppler filtering or the Fast Fourier Transform (FFT). Monopulse angle measurement commonly employs the amplitude comparison method, where the amplitude of signals in the sum and difference channels is compared. The resulting ratio is mapped onto the antenna pattern’s angle-discrimination curve to estimate the target’s direction of arrival.

#### 2.1.2. Data Processing

While the signal processing of monopulse radar effectively enhances target detection performance, it can still lead to the identification of false alarm targets. The purpose of the data processing is to achieve stable target detection, eliminate false alarms, and ensure accurate target tracking. The data processing workflow includes constant CFAR detection, track initiation, track association, and tracking filtering.

CFAR detection is employed to automatically detect targets from the data of MTD process while maintaining a constant false alarm rate in complex environments, thereby enhancing detection reliability. Track initiation, track association, and tracking filtering ensure stable target tracking.

### 2.2. Received Signal Model

#### 2.2.1. Radar Echo Signal

When the radar transmits a linear frequency-modulated (LFM) signal of the following form:

(1) $$S(t)=A*rect[\frac{t}{T}]e^{j2\pi (f_{0}t+\frac{1}{2}\mu t^{2})}$$

where A is the signal amplitude; $f_{0}$ is the radar signal carrier frequency and μ is the chirp rate; rect[∙] is the gate function; and T denotes the PRI. For an extended target, it can be modeled as a multi-scatterer model comprising K scattering points, with its range-domain scattering characteristic function given by [32]:

(2) $$CF(t)=\sum_{k=1}^{K}\sqrt{\sigma_{k}}e^{j2\pi \phi_{k}}\delta (t-\tau_{k})$$

where $\sqrt{\sigma_{k}}$, $\phi_{k}$, and $\tau_{k}$ represent the amplitude, initial phase, and delay of echo signal corresponding to the k-th scatterer, respectively.

The echo signal in the radar’s optical region is the convolution of the transmitted signal and the scattering characteristic function, and it can be expressed as [32]:

(3) $$\begin{array}{ll} S_{r}(t) & =S(t)⊗CF(t)\\ & =A\sum_{k=1}^{K}\sqrt{\sigma_{k}}e^{j2\pi \phi_{k}}rect[\frac{t-2R_{k}/c}{T}]\\ & \cdot \exp\{j\pi \mu {\left(t-2R_{k}/c\right)}^{2}\}\\ & \cdot \exp\{j2\pi (f_{0}+f_{kd})(t-R_{k}/c)\} \end{array}$$

where $R_{k}$ denotes the radial range of the k-th scatterer, $v_{k}$ is the relative radial velocity between the radar and the k-th scatterer, and $f_{kd}=2v_{k}f_{0}/c$ is the Doppler frequency of the k-th scatterer; *c* is the speed of light.

The signal received by the radar is then expressed as:

(4) $$x(t)=S_{r}(t)+N(t)+C(t)+J(t)$$

where $S_{r}(t)$ is the target echo signal; N(t) is the noise, which includes both internal receiver noise, as well as antenna and external environmental noise; and C(t) and J(t) are the clutter and jamming, respectively.

In a monopulse radar system, four beams are typically used to form the antenna radiation pattern. The pattern functions of the four beams are denoted as $F_{A}(\Delta \theta \;\Delta \varphi )$, $F_{B}(\Delta \theta ,\Delta \varphi )$, $F_{C}(\Delta \theta ,\Delta \varphi )$, and $F_{D}(\Delta \theta ,\Delta \varphi )$, where $\Delta \theta =\theta_{t}-\theta_{p},\;\Delta \varphi =\varphi_{t}-\varphi_{p}$, and $\left(\theta_{t},\varphi_{t}\right)$ are, respectively, the azimuth and elevation of the target. $\left(\theta_{p},\varphi_{p}\right)$ are the azimuth and elevation beam squint angle between the antenna’s Line of Sight (LOS) and the rotation axis, respectively. The beam squint angle is defined as the angular between the beam axis and tracking axis [33]. The sum of signals from beams A, B, C, and D is denoted as sum signal $\sum \left(\theta_{t},\varphi_{t}\right)$. The difference between the signals from beams A and C and from beams B and D corresponds to the elevation difference signal $\Delta_{el}(\theta_{t},\varphi_{t})$, while the difference between the signals from beams A and B and from beams C and D corresponds to the azimuth difference signal $\Delta_{az}(\theta_{t},\varphi_{t})$. Therefore, the sum and difference singles can be expressed, respectively, as [34]:

(5) $$\sum \left(\theta_{t},\varphi_{t}\right)=x(t)[F_{A}(\Delta \theta ,\Delta \varphi )+F_{B}(\Delta \theta ,\Delta \varphi )+F_{C}(\Delta \theta ,\Delta \varphi )+F_{D}(\Delta \theta ,\Delta \varphi )]$$

(6) $$\Delta_{el}(\theta_{t},\varphi_{t})=x(t)[F_{A}(\Delta \theta ,\Delta \varphi )+F_{C}(\Delta \theta ,\Delta \varphi )-(F_{B}(\Delta \theta ,\Delta \varphi )+F_{D}(\Delta \theta ,\Delta \varphi ))]$$

(7) $$\Delta_{\mathrm{az}}(\theta_{t},\varphi_{t})=x(t)[F_{A}(\Delta \theta ,\Delta \varphi )+F_{B}(\Delta \theta ,\Delta \varphi )-(F_{C}(\Delta \theta ,\Delta \varphi )+F_{D}(\Delta \theta ,\Delta \varphi ))].$$

In the above equations, sum signal is used to estimate the range and velocity of the target. The azimuth and elevation of the target are determined by calculating the ration of the sum signal and azimuth, as well as elevation difference signal.

#### 2.2.2. Jamming Signals

##### Suppression Jamming

Suppression jamming, also known as masking jamming, is a traditional form of signal suppression. Typically, it is implemented by modulating Gaussian white noise. It can be categorized into four forms based on the modulation method: radio frequency (RF) noise jamming, amplitude-modulated noise jamming, frequency-modulated noise jamming, and phase-modulated noise jamming. The specific expressions for these four types of jamming are outlined below.

The expression for RF noise jamming is given by [35]:

(8) $$J(t)=U_{n}(t)e^{j(\omega_{j}t+\varphi (t))}$$

where $U_{n}(t)$ follows a Rayleigh distribution and the phase φ(t) follows a uniform distribution over the interval $\left[0,\left.2\pi \right]\right.$, independent of $U_{n}(t)$. The carrier frequency $\omega_{j}$ is constant and much greater than the bandwidth of the noise signal J(t).

The expression for amplitude-modulated noise jamming is [36]:

(9) $$J(t)=\left[U_{0}+U_{n}(t)\right]e^{j(\omega_{j}t+\varphi (t))}$$

where the modulating noise $U_{n}(t)$ is a zero-mean, stationary random process with variance $\sigma_{n}^{2}$, distributed over the interval $\left[-U_{0},\;\left.\infty \right)\right.$. φ follows a uniform distribution over the interval $\left[0,\left.\;2\pi \right]\right.$, independent of $U_{n}(t)$. $U_{0}$ and $\omega_{j}$ are constants.

The expression for frequency-modulated noise jamming is [37]:

(10) $$J\left(t\right)=U_{j}e^{j(\omega_{j}t+2\pi K_{FM}\int_{0}^{t}u_{n}\left(t^{\prime }\right)dt^{\prime }+\varphi )}$$

where $U_{j}$ is the jamming signal amplitude and $K_{FM}$ represents the frequency modulation slope; $u_{n}(t)$ is a stationary random noise with zero mean and variance $\sigma_{n}^{2}$, known as the modulation noise. The initial phase φ is uniformly distributed over [0,2π] and is independent of $u_{n}(t)$.

The expression for phase-modulated noise jamming is [38]:

(11) $$J\left(t\right)=U_{j}e^{j(\omega_{j}t+\varphi (t)+\phi )}$$

where φ(t) is a zero-mean, stationary random process over the interval $\left[-\pi ,\pi \right]$.

##### Deceptive Jamming

Deceptive jamming involves generating false target echo signals that jam the radar’s detection and tracking system, preventing the radar from correctly detecting the true target or measuring the parameters of the real target. Multiple false targets are used to deceive pulse radar range measurement information, typically achieved by storing radar-transmitted signals and modulating them in time delay and amplification for retransmission.

Let R denote the radial range of the real target. The time delay of the radar’s echo pulse envelope is given by:

(12) $$\tau_{r}=2R/c.$$

The time delay of the false target’s echo pulse envelope is:

(13) $$\tau_{f}=2R_{f}/c.$$

When $\left|R_{f}-R\right|>\Delta R$ is met, a false target is formed. $\tau_{f}$ typically consists of two components [39]:

(14) $$\left\{\begin{array}{l} \tau_{f}=\tau_{f_{0}}+\Delta \tau_{f}\\ \tau_{f_{0}}=2R_{J}/c \end{array}\right.$$

where $R_{J}$ is the radial range between the radar and the jammer, $\tau_{f_{0}}$ is the transmission delay caused by $R_{J}$, and $\Delta \tau_{f}$ is the repeater delay after the jammer receives the radar signal.

Depending on the repeater delay, false targets can be classified as either near or far false targets. A near false target refers to a target that is closer to the radar than the real target, while a far false target is one that is farther from the radar than the real target. Near false targets are typically formed in the next PRI, and their effectiveness depends on the stability of the radar’s RF and PRI. Far false targets are formed within a PRI.

**Figure 2:**
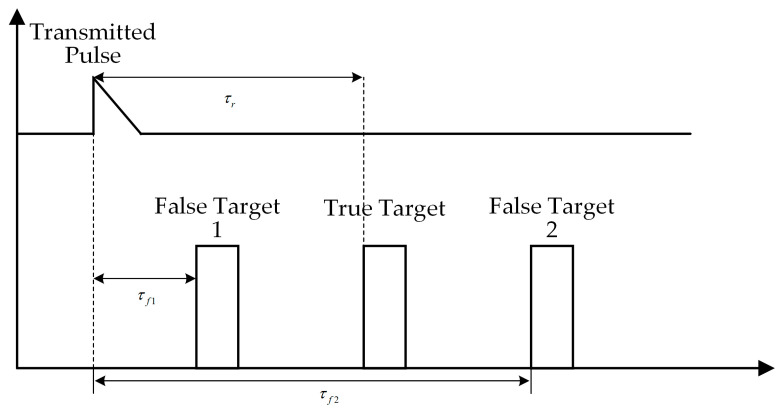
False target jamming in pulse radar range detection.

As shown in Figure 2, False Target 1 is a near false target and False Target 2 is a far false target. $\tau_{r}$ is the real target echo delay, while $\tau_{f_{1}}$ and $\tau_{f_{2}}$ represent the echo delay of False Target 1 and False Target 2, respectively.

It can be observed that the fidelity of the jamming signal, radar PRI, and RF stability all affect the effectiveness of the jamming.

## 3. Space Slicing Simulation System Construction

### 3.1. Overview of Space Slicing Method

In the simulation process of a monopulse radar under a noisy environment, a substantial number of complex operations are required, which significantly increase the simulation time and impact the real-time performance of the simulation system. This paper presents a space slice-based method, dividing a given distance range into discrete slices at a certain granularity. In each slice, the jamming types are considered. The processing results of each slice for different jamming types provide the target’s range, velocity, and angles data under different radar operation states.

**Figure 3:**
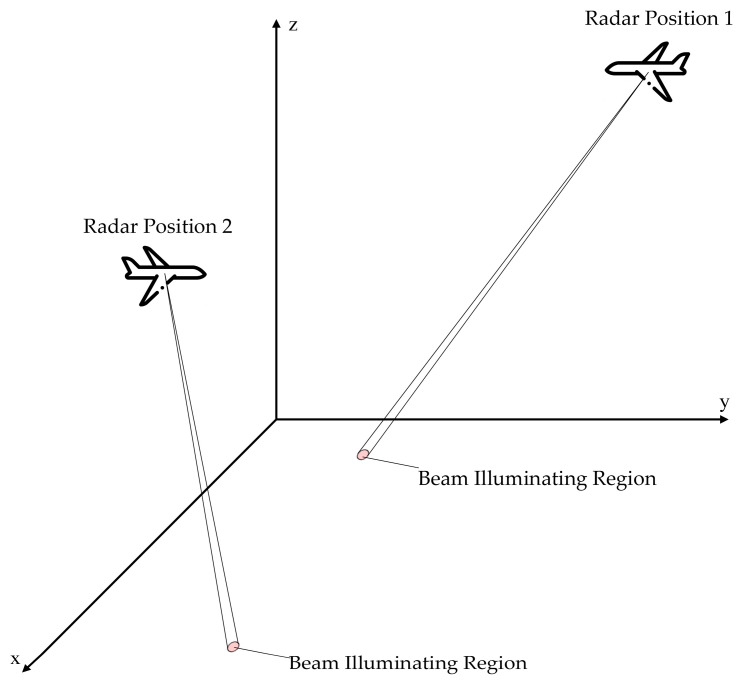
Radar illumination schematic.

As illustrated in Figure 3, Radar Position 1 and Radar Position 2 represent different spatial positions. The radar illuminates different regions by means of beam scanning. From the figure, the two circles represent the illuminating range of the two beam positions or beams. The corresponding range and angles data in this circle is regarded as a slice. Due to the effect of the jamming, different jamming types are considered in each slice.

**Figure 4:**
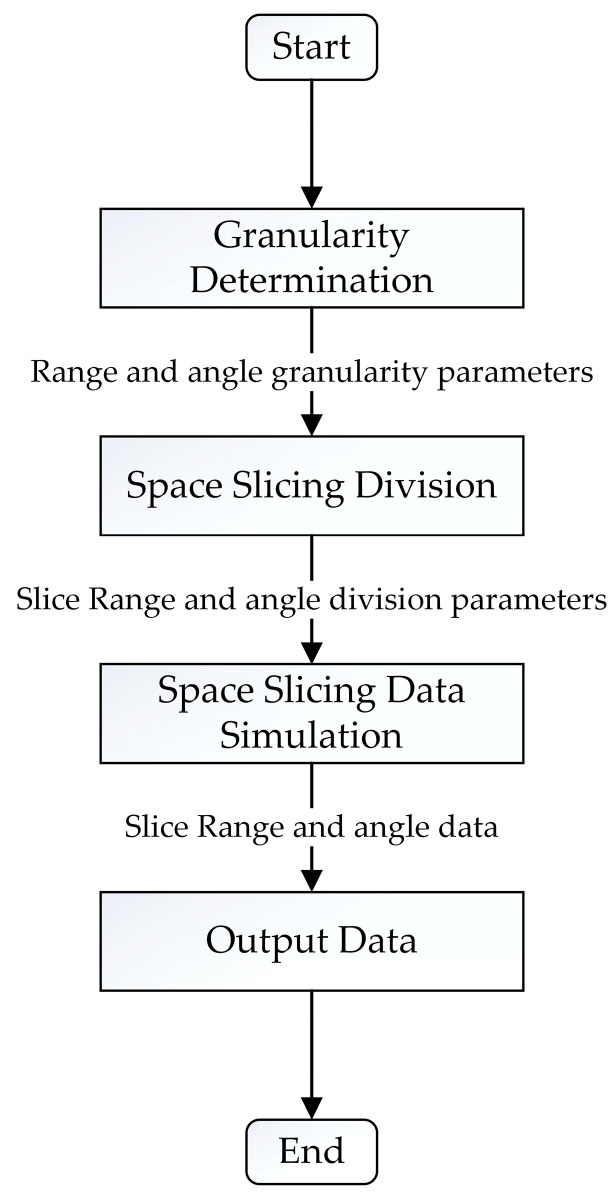
Space slicing workflow diagram.

The space slicing workflow diagram is shown in Figure 4; radar operating state, jamming type, target radial range, and velocity, as well as beam scanning range, are used to determine the granularity. After this, space slicing division is executed with these granularity parameters. Space slicing simulation, including signal and data processing, is performed using the division parameters for each radar operating state. Finally, the space slicing data are produced.

### 3.2. Space Slice Method for Different Radar Operating States

A monopulse radar has four distinct operating states: search, narrow search, acquisition, and track. In the search state, the antenna scans over a wide range using different beam positions to locate targets. When a target is detected, the radar determines the radial range and velocity, as well as the beam position at which the target is located.

The narrow search state operates similarly to the search state but covers a smaller range for target detection. In the acquisition state, the radar confirms the target by performing a series of smaller beam scanning over a limited range. If a target is consistently detected at a particular beam position throughout multiple scans and the target’s physical characteristics closely match pre-defined target information, the target can be confirmed.

The track state is used for stable target tracking. In this state, the radar beam no longer performs scans but instead continuously illuminates the target. During this process, the radar continuously outputs the target’s range, velocity, and angle.

#### 3.2.1. Space Slicing for Search State

**Figure 5:**
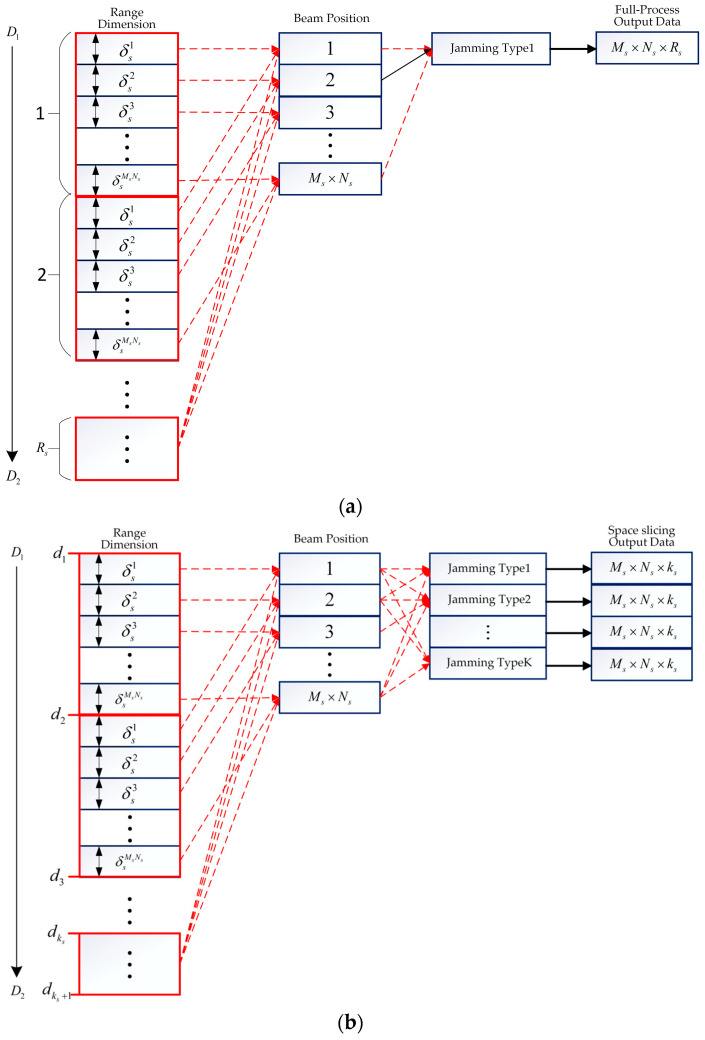
Full-process and space slicing process for search state. (**a**) Full-process process for search state. (**b**) Space slicing process for search state.

In the search state, assume that the target is located at the range $\left(D_{1},D_{2}\right)$. The azimuth and elevation beams scan, respectively, within $\left(-\theta_{s},\theta_{s}\right)$ and $\left(-\varphi_{s},\varphi_{s}\right)$. There are $M_{s}$ and $N_{s}$ azimuth and elevation beam positions during a single search round, respectively. The dwell time for each scan beam position is denoted as $T_{s}$, and the radial velocity of the target is $v_{s}$. Then, the range between radar and target will be shortened by $\Delta d_{s}=M_{s}N_{s}T_{s}v_{s}$ after a search round when the radar is moving towards the target. The full process and space slicing process for search state are shown in Figure 5. The left-most column denotes the range dimension data distributed in $\left(D_{1},D_{2}\right)$, with each square box representing the distance variation for one beam position. The second column denotes the beam position. The red arrowhead depicts the relationship of the beam position and distance variation. The third column represents the jamming type, which implies the radar works on the jamming scenarios. The last column is the dimension of the output data.

The full-process process for search state is illustrated in Figure 5a, where radar searches the target located at range $\left(D_{1},D_{2}\right)$ after *Rs* search rounds. The distance variation for each beam position is denoted as $\delta_{s}^{i},\;i\in [1,M_{s}N_{s}]$. From the figure, under a jamming type, $M_{s}\times N_{s}\times R_{s}$ output data is obtained. Once the electromagnetic environment changes, the full-process simulation is performed again. This results in the huge computational complexity in the radar anti-jamming evaluation.

If all the jamming types are known, the monopulse radar processing results can be pre-produced under jamming environment. And, then, the anti-jamming evaluation results are obtained quickly by choosing the appropriate slice data according to the demands. The space slicing process is illustrated in Figure 5b. Slicing the search range $\left(D_{1},D_{2}\right)$ at a grid size of $\Delta d_{s}$, $k_{s}=\left\lceil (D_{2}-D_{1})/\Delta d_{s}\right\rceil$ slices will be obtained. The intervals are denoted by $d_{1},d_{2},\ldots ,d_{k_{s}},d_{k_{s}+1}$ in ascending order. In the figure, $k_{s}=R_{s}$ slices can be performed parallel, which improves the computational efficiency. It is noted that all kinds of jamming can be added in the echoes to generate different target detection results, which can evaluate the radar anti-jamming performance sufficiently. In contrast, the simulation is performed for each jamming type with the full-process method. This is the reason why the space slice is superior to the full-process method.

#### 3.2.2. Space Slicing for Narrow Search State

The narrow search state is performed when the radar loses the target. In order to capture the target quickly, the searching range and beam positions for the narrow search state are limited within a relatively small zone, which is widely used in the practical application [40]. In this paper, comparing the search state, the spatial scanning range is smaller and the search intervals are finer in the narrow search state. The slicing process is the same as that for search state.

#### 3.2.3. Space Slicing for Acquisition State

**Figure 6:**
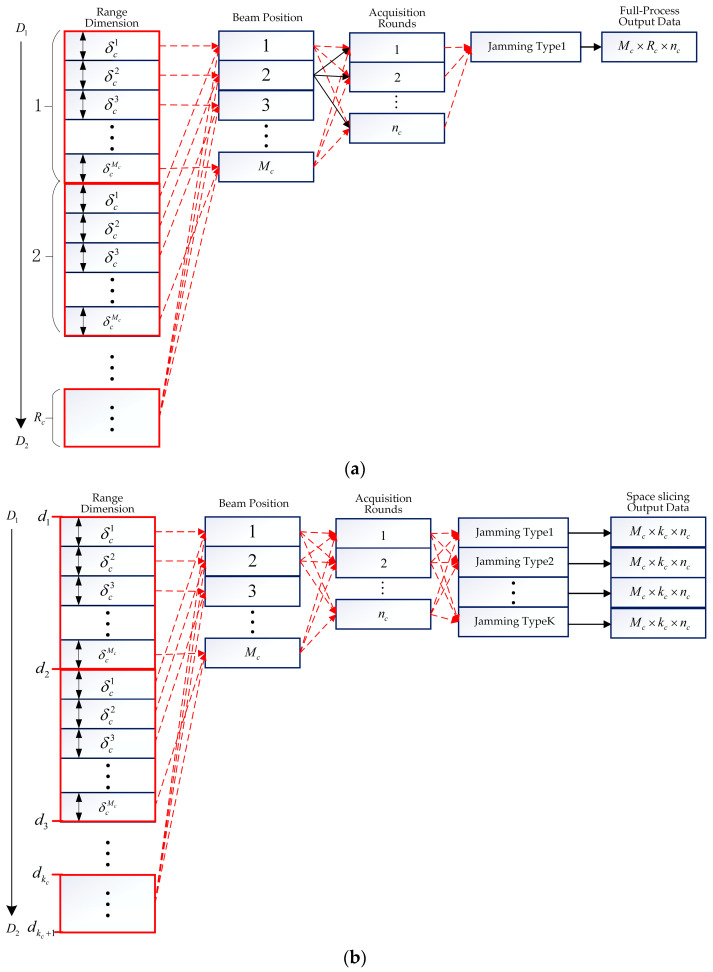
Full-process and space slicing process for acquisition state. (**a**) Full-process process for acquisition state. (**b**) Space slicing process for acquisition state.

In the acquisition state, the radar searches a smaller angle range, covering a total of $M_{c}$ beam positions. The antenna scanning needs to be performed $n_{c}$ rounds to confirm the target, which is depicted in Figure 6. Assume that radar takes a total time of $T_{c}$ in every scanning round. If the radial velocity of target is $v_{c}$, then the target’s range change during one acquisition cycle is approximately $\Delta d_{c}=M_{c}T_{c}v_{c}$. From Figure 6a, in the acquisition state, the radar must detect the target multiple times to confirm the real target, which is different with the search state. In Figure 6b, the target’s range is then divided into intervals at a granularity of $\Delta d_{c}$, represented by segments $d_{1},d_{2},\ldots ,d_{k_{c}},d_{k_{c}+1}$ in ascending order, where $k_{c}=\left\lceil (D_{2}-D_{1})/\Delta d_{c}\right\rceil$. Similarly, slices data can be obtained under all kinds of jamming in the acquisition state. It is noted that the distance variation corresponding to each beam position $\delta_{c}^{i},\;i\in [1,M_{c}]$ is usually smaller than that for search state. This is because the beam position in the acquisition state is narrower.

#### 3.2.4. Space Slicing for Track State

**Figure 7:**
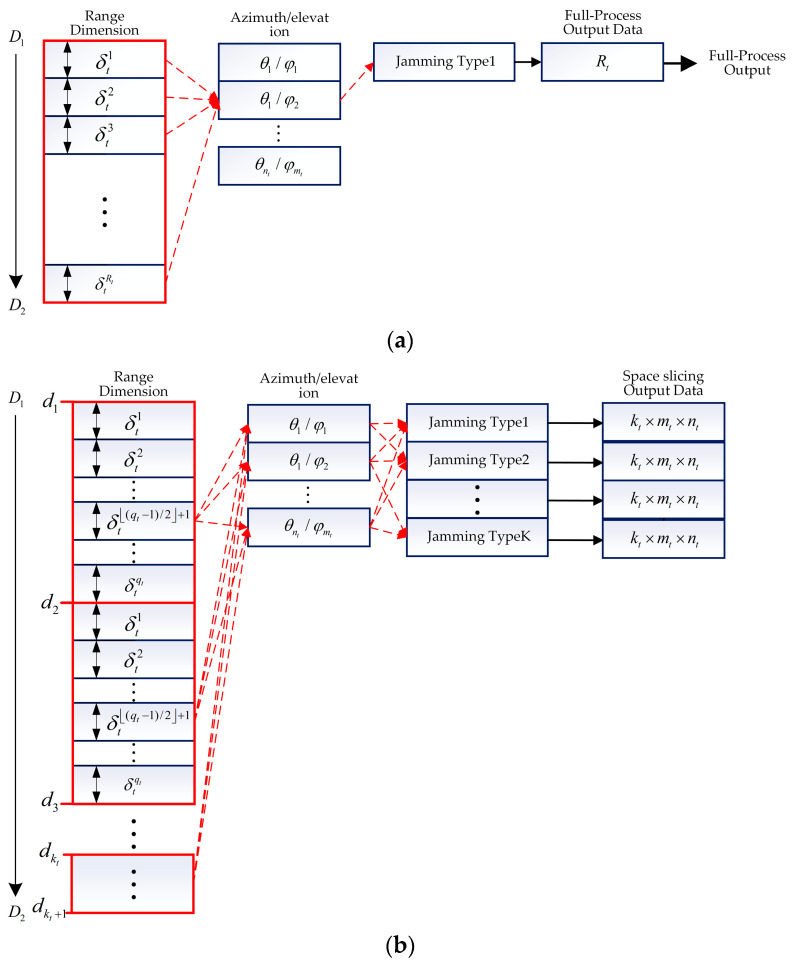
Full-process and space slicing process for track state. (**a**) Full-process process for track state. (**b**) Space slicing process for track state.

In the track state, the narrower pulse and shorter PRI are usually used in the radar system, which leads to the higher data rate. Therefore, the slice strategy should be changed. Assuming that $v_{t}^{\prime }$ represents the target’s velocity and $\Delta D_{t}$ denotes range bin, $T_{t}$ is the coherent processing interval (CPI). The range walk does not happen in $q_{t}$ CPIs with $q_{t}=\left\lceil \Delta D_{t}/({v^{\prime }}_{t}T_{t})\right\rceil$, where $\left\lceil ∙\right\rceil$ is a round up to an integer value of the function. In Figure 7a, $\delta_{t}^{i},\;i\in [1,R_{t}]$ represents the range variation corresponding to one CPI, with $R_{t}=\left\lceil \left(D_{2}-D_{1})/(T_{t}v_{t}\right)\right\rceil$, where $v_{t}$ is the radial velocity of the target. In the track state, the monopulse tracking technique is used to obtain fine angular measurement. For the full-process simulation, monopulse tracking technique is performed within the azimuth space $\left[\theta_{1},\theta_{n_{t}}\right]$ and elevation space $\left[\varphi_{1},\varphi_{m_{t}}\right]$. Finally, $R_{t}$ radar processing results data is obtained for one jamming type. It is noted that only one pair of azimuth and elevation is obtained for a real-time simulation in the full-process simulation.

In order to improve the computational efficiency, the range variation of $q_{t}$ CPIs are regarded as a slice, with the center of each CPI representing the slice, i.e., $\delta_{t}^{\left\lfloor (q_{t}-1)/2\right\rfloor +1}$ represents the $q_{t}$ CPI. This can be seen in Figure 7b. In this situation, the target range is divided into intervals at a granularity of $\Delta d_{t}=q_{t}T_{t}v_{t}$, represented by segments in increasing order $d_{1},d_{2},\ldots ,d_{k_{t}},d_{k_{t}+1}$, where $k_{t}=\left\lceil (D_{2}-D_{1})/\Delta d_{t}\right\rceil$. Finally, $k_{t}\times m_{t}\times n_{t}$ output data are obtained for every jamming type.

It is noted that the center of each CPI is used to represent the slice in the track state, which decreases the data rate. On the other hand, this reduces the range accuracy, compared with the full-process method. This will be discussed in Section 3.3.

#### 3.2.5. Monopulse Radar Simulation Based on the Slice Data Selection

As demonstrated above, the slice dataset can be pre-constructed. In practical applications, the radar operates in four different states: search, acquisition, track, and narrow search. However, the slice dataset is divided into different operational states, and appropriate slice data must be selected to restore the radar’s operational process.

**Figure 8:**
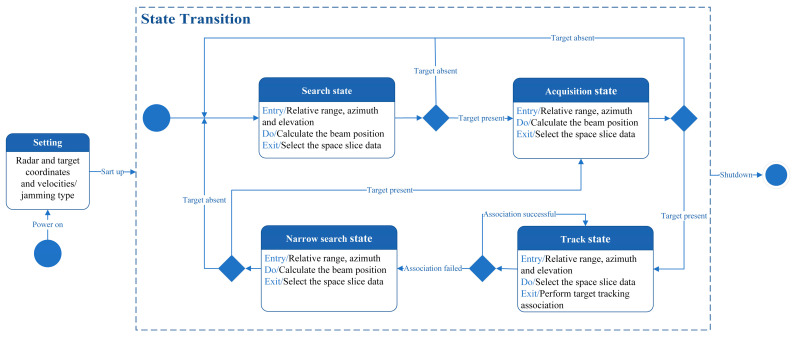
Simulation analysis based on space slicing data.

The state diagram is shown in Figure 8. In Figure 8, the relative range, velocity, azimuth, and elevation between the radar and the target are calculated using the coordinates and velocity vectors of both the radar and the target. The algorithm begins in the search state, where slice data is selected based on the obtained beam position. If the slice data does not contain target information, it indicates a radar anti-jamming failure, and the radar continues searching for the target; otherwise, it transitions to the acquisition state. Similarly, in the acquisition state, slice data is selected based on the calculated beam position. If the slice data does not contain target information, it indicates a radar target capture failure, and the radar continues searching for the target; otherwise, it transitions to the track state. In the track state, slice data is used for target association. If the association is successful, the radar remains in the track state; otherwise, it transitions to the narrow search state. The narrow search state is similar to the search state but with finer search precision. If the slice data does not contain target information, it transitions back to the search state; otherwise, it transitions to the interception state. This process continues until the radar is powered off.

### 3.3. Error Analysis of Space Slicing

In space slicing, the target’s range and angles are divided into slices at different granularities for different radar operational states. During simulation, slices closest to the actual radar position are selected to approximate the radar’s output. If these slices accurately represent multiple positions along the radar’s trajectory, the slicing method is deemed analytically valuable.

#### 3.3.1. Error Analysis of Space Slicing for Search, Acquisition, and Narrow Search State

(1) When there is no jamming or jamming fails, the target is located in only one beam position, producing the target’s range, velocity, and beam position, while all other beam positions yield no output. The data results from this slice are the same as those of full-process simulation since the echo in the same beam position is used to be processed.
(2) When suppressive jamming succeeds, the target cannot be detected in all beam positions. As a result, no target information is outputted, which indicates the radar does not detect or identify the target in search, acquisition, or narrow search mode successfully. This is the same as the full-process simulation. When deceptive jamming succeeds, using slicing method, the radar outputs data results of true and false target, which is also the same as the full-process simulation.

#### 3.3.2. Error Analysis of Space Slicing for Track State

In the track state, the beam of the radar antenna is pointed at the target and only the range and angles are sliced. Compared with the full-process simulation, the target’s range, velocity, and angles from slices are erroneous since the center CPI of $q_{t}$ CPIs is used to represent this slice. This is analyzed below.

**Figure 9:**
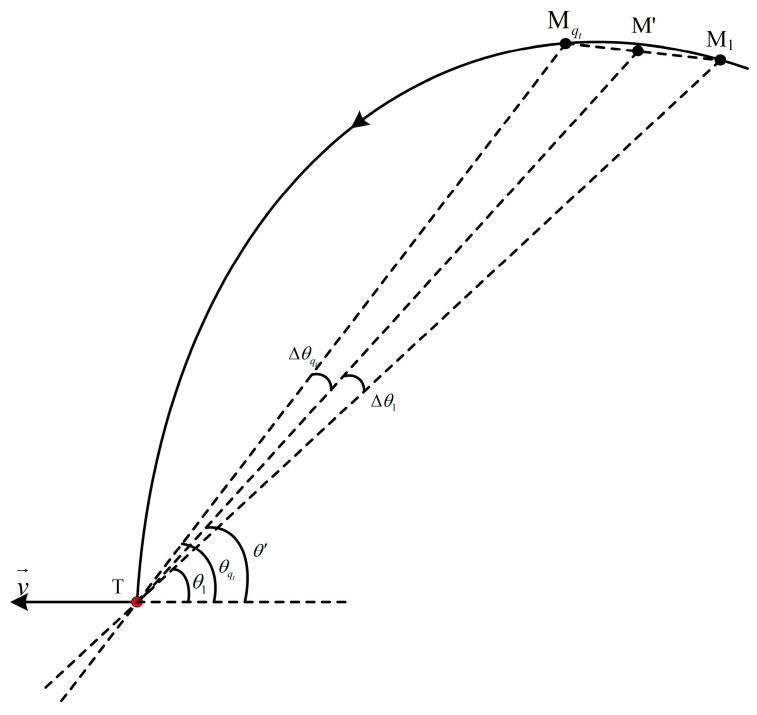
Schematic of azimuthal motion trajectory in track state.

Consider the radar and target are moving in Figure 9. Assume that, within $q_{t}$ consecutive CPIs, the radar moves from spatial position $M_{1}$ to $M_{q_{t}}$, with T denoting the target position. $M^{\prime }$ is the midpoint between $M_{1}$ and $M_{q_{t}}$. $M_{\alpha }$ for $a=1,2,\ldots ,q_{t}$ is the α-th point from $M_{1}$ to $M_{q_{t}}$. The beam always points to the target T when the radar moves from position $M_{1}$ to $M_{q_{t}}$. The angles $\theta_{1}$ and $\theta_{q_{t}}$ are the respective azimuths of position $M_{1}$ and $M_{q_{t}}$, while $\theta^{\prime }$ is the azimuth of position $M^{\prime }$. $\Delta \theta_{1}$ and $\Delta \theta_{q_{t}}$ are the squint angles between the line $M_{1}T$ and $M^{\prime }T$, as well as $M_{q_{t}}T$ and $M^{\prime }T$, respectively.

With the space slicing method, the angle measurement of position $M^{\prime }$ is used to replace the $q_{t}$ angle measurements from position $M_{1}$ to $M_{q_{t}}$, resulting in angle measurement bias. According to the cosine theorem, the angle measurement bias $\Delta \theta_{\alpha }$ of position $M_{\alpha }$ is expressed as

(15) $$\Delta \theta_{\alpha }=\arccos\left(\frac{{\left|{\mathrm{TM}}_{\alpha }\right|}^{2}+{\left|{\mathrm{TM}}^{\prime }\right|}^{2}-{\left|M_{\alpha }M^{\prime }\right|}^{2}}{2\left|{\mathrm{TM}}_{\alpha }\right|\left|{\mathrm{TM}}^{\prime }\right|}\right).$$

From Figure 9, $\left|M_{\alpha }M^{\prime }\right|=\gamma T_{t}v_{t}^{"}$ and $\left|{\mathrm{TM}}^{\prime }\right|-\left|{\mathrm{TM}}_{\alpha }\right|=\gamma \Delta d_{t}/q_{t}$, with $\gamma =\left|\alpha -\left\lceil q_{t}/2\right\rceil \right|$, where $\left|∙\right|$ denotes the mod operator. $v_{t}^{\prime \prime }$ represents the velocity of the radar. Then, (15) can be rewritten as

(16) $$\Delta \theta_{\alpha }=\arccos\left(\frac{2{\left|{\mathrm{TM}}_{\alpha }\right|}^{2}+\frac{2\gamma }{q_{t}}\left|{\mathrm{TM}}_{\alpha }\right|\Delta d_{t}+\left({\left(\frac{\gamma }{q_{t}}\right)}^{2}-{\left(\frac{\gamma v_{t}^{\prime \prime }}{q_{t}v_{t}}\right)}^{2}\right)\Delta d_{t}^{2}}{2{\left|{\mathrm{TM}}_{\alpha }\right|}^{2}+\frac{2\gamma }{q_{t}}\left|{\mathrm{TM}}_{\alpha }\right|\Delta d_{t}}\right).$$

The angle measurement bias reaches the maximum at points $M_{1}$ or $M_{q_{t}}$. The angle measurement bias $\Delta \theta_{1}$ and $\Delta \theta_{q_{t}}$ are given by

(17) $$\Delta \theta_{1}=\arccos\left(\frac{{\left|{\mathrm{TM}}_{1}\right|}^{2}+{\left|{\mathrm{TM}}^{\prime }\right|}^{2}-{\left|M_{1}M^{\prime }\right|}^{2}}{2\left|{\mathrm{TM}}_{1}\right|\left|{\mathrm{TM}}^{\prime }\right|}\right)\;\Delta \theta_{q_{t}}=\arccos\left(\frac{{\left|{\mathrm{TM}}_{q_{t}}\right|}^{2}+{\left|{\mathrm{TM}}^{\prime }\right|}^{2}-{\left|M_{2}M^{\prime }\right|}^{2}}{2\left|{\mathrm{TM}}_{q_{t}}\right|\left|{\mathrm{TM}}^{\prime }\right|}\right)$$

Therefore, the angular bias of space slicing can be expressed as $\Delta \theta =\max(\Delta \theta_{1},\Delta \theta_{q_{t}})$. From Figure 9, $\left|M_{q_{t}}M^{\prime }\right|=\left|M_{1}M^{\prime }\right|=q_{t}T_{t}v_{t}^{"}/2$ and $\left|{\mathrm{TM}}^{\prime }\right|-\left|{\mathrm{TM}}_{1}\right|=\left|{\mathrm{TM}}_{q_{t}}\right|-\left|{\mathrm{TM}}^{\prime }\right|=\Delta d_{t}/2$. Then, (17) can be rewritten as

(18) $$\Delta \theta_{1}=\arccos\left(\frac{2{\left|{\mathrm{TM}}_{1}\right|}^{2}+\left|{\mathrm{TM}}_{1}\right|\Delta d_{t}+\left(\frac{1}{4}-{\left(\frac{v_{t}^{\prime \prime }}{2v_{t}}\right)}^{2}\right)\Delta d_{t}^{2}}{2{\left|{\mathrm{TM}}_{1}\right|}^{2}+\left|{\mathrm{TM}}_{1}\right|\Delta d_{t}}\right)\;\Delta \theta_{q_{t}}=\arccos\left(\frac{2{\left|{\mathrm{TM}}^{\prime }\right|}^{2}+\left|{\mathrm{TM}}^{\prime }\right|\Delta d_{t}+\left(\frac{1}{4}-{\left(\frac{v_{t}^{\prime \prime }}{2v_{t}}\right)}^{2}\right)\Delta d_{t}^{2}}{2{\left|{\mathrm{TM}}^{\prime }\right|}^{2}+\left|{\mathrm{TM}}^{\prime }\right|\Delta d_{t}}\right).$$

Let $f(\Delta d_{t})=\left(2{\left|{\mathrm{TM}}_{1}\right|}^{2}+\left|{\mathrm{TM}}_{1}\right|\Delta d_{t}+{\left(\frac{1}{4}-\frac{v_{t}^{\prime \prime }}{v_{t}}\right)}^{2}\Delta d_{t}^{2}\right)/2{\left|{\mathrm{TM}}_{1}\right|}^{2}+\left|{\mathrm{TM}}_{1}\right|\Delta d_{t}$, then $\partial f(\Delta d_{t})/\partial \Delta d_{t}=\left(\frac{1}{4}-\frac{{v^{\prime \prime }}_{t}^{2}}{4v_{t}^{2}}\right)\left(2\left|{\mathrm{TM}}_{1}\right|+\frac{\Delta d_{t}}{2}\right)\Delta d_{t}/\left(2\left|{\mathrm{TM}}_{1}\right|{\left(\left|{\mathrm{TM}}_{1}\right|+\frac{\Delta d_{t}}{2}\right)}^{2}\right)$. Since $\left|v_{t}^{\prime \prime }\right|\geq \left|v_{t}\right|$, $\partial f(\Delta d_{t})/\partial \Delta d_{t}\leq 0$. Therefore, $f(\Delta d_{t})$ decreases with the increment of $\Delta d_{t}$, which indicates that $\Delta \theta_{1}$ increases with the increment of $\Delta d_{t}$. Similarly, $\Delta \theta_{q_{t}}$ has the same characteristic.

If $\left|M_{1}M_{q_{t}}\right|$ is given, the angular variation reaches the maximum when $M_{1}M_{q_{t}}⊥M_{q_{t}}T$. Assuming the radar enters the track state at a range of $R_{1}$ and the radar moves a distance $\Delta d_{t}$ within $q_{t}$ CPIs (i.e., over $q_{t}T_{t}$ seconds), the maximum angular error is $\Delta \theta =\arctan(\Delta d_{t}/2R_{1})$. However, in practical scenarios, the trajectory rarely satisfies $M_{1}M_{q_{t}}⊥M_{q_{t}}T$, meaning that the actual angular variation is significantly smaller than the theoretical maximum.

As shown in Figure 9, assume the target’s velocity vector is $\vec{v}$. The azimuth $\theta_{\alpha }$ of the position $M_{\alpha }$ can be expressed as $\theta^{\prime }\pm \Delta \theta_{\alpha }$. Let $\Delta v_{\alpha }$ represent the radial velocity measurement bias from $M_{\alpha }$ to $M^{\prime }$

(19) $$\Delta v_{\alpha }=\left|\vec{v}\right|\left|\cos\theta_{\alpha }-\cos\theta^{\prime }\right|.$$

Substituting (16) into (19) gives

(20) $$\Delta v_{\alpha }=\left|\vec{v}\right|\left|\cos\left(\theta^{\prime }\pm \arccos\left(\frac{2{\left|{\mathrm{TM}}_{\alpha }\right|}^{2}+\frac{2\gamma }{q_{t}}\left|{\mathrm{TM}}_{\alpha }\right|\Delta d_{t}+\left({\left(\frac{\gamma }{q_{t}}\right)}^{2}-{\left(\frac{\gamma v_{t}^{\prime \prime }}{q_{t}v_{t}}\right)}^{2}\right)\Delta d_{t}^{2}}{2{\left|{\mathrm{TM}}_{\alpha }\right|}^{2}+\frac{2\gamma }{q_{t}}\left|{\mathrm{TM}}_{\alpha }\right|\Delta d_{t}}\right)\right)-\cos\theta^{\prime }\right|.$$

Similarly, the velocity measurement bias reaches maximum at points $M_{1}$ or $M_{q_{t}}$; then, $\theta_{1}=\theta^{\prime }-\Delta \theta_{1}$ and $\theta_{q_{t}}=\theta^{\prime }+\Delta \theta_{q_{t}}$. Let $\Delta v_{1}$ and $\Delta v_{q_{t}}$ represent the radial velocity bias from $M_{1}$ to $M^{\prime }$ and $M^{\prime }$ to $M_{q_{t}}$, respectively, which are given by:

(21) $$\Delta v_{1}=\left|\vec{v}\right|\left|\cos(\theta^{\prime }-\Delta \theta_{1})-\cos\theta^{\prime }\right|\;\Delta v_{q_{t}}=\left|\vec{v}\right|\left|\cos(\theta^{\prime }+\Delta \theta_{q_{t}})-\cos\theta^{\prime }\right|.$$

From the above equation, the maximum velocity measurement bias of space slicing can be expressed as $\Delta v=\max(\Delta v_{1},\Delta v_{q_{t}})$, which depends on the variation in $\Delta \theta_{1}$ and $\Delta \theta_{q_{t}}$. If $\Delta \theta_{1}$, $\Delta \theta_{q_{t}}$, and the variation in $\vec{v}$ is very small within $q_{t}$ CPIs, Δv is almost unchanged. Then, the velocity from slices is the same as that of full-process simulation.

Substituting (18) into (21) yields:

(22) $$\Delta v_{1}=\left|\vec{v}\right|\left|\cos\left(\theta^{\prime }-\arccos\left(\frac{2{\left|{\mathrm{TM}}_{1}\right|}^{2}+\left|{\mathrm{TM}}_{1}\right|\Delta d_{t}+\left(\frac{1}{4}-{\left(\frac{v_{t}^{\prime \prime }}{2v_{t}}\right)}^{2}\right)\Delta d_{t}^{2}}{2{\left|{\mathrm{TM}}_{1}\right|}^{2}+\left|{\mathrm{TM}}_{1}\right|\Delta d_{t}}\right)\right)-\cos\theta^{\prime }\right|\;\Delta v_{q_{t}}=\left|\vec{v}\right|\left|\cos\left(\theta^{\prime }+\arccos\left(\frac{2{\left|{\mathrm{TM}}^{\prime }\right|}^{2}+\left|{\mathrm{TM}}^{\prime }\right|\Delta d_{t}+\left(\frac{1}{4}-{\left(\frac{v_{t}^{\prime \prime }}{2v_{t}}\right)}^{2}\right)\Delta d_{t}^{2}}{2{\left|{\mathrm{TM}}^{\prime }\right|}^{2}+\left|{\mathrm{TM}}^{\prime }\right|\Delta d_{t}}\right)\right)-\cos\theta^{\prime }\right|.$$

Based on the analysis of (18), $\Delta v_{1}$ and $\Delta v_{q_{t}}$ increase with the increment of $\Delta d_{t}$.

For range tracking, if the target’s range over several CPIs do not fall in the range predicted gate, the target is considered lost, prompting the radar to transition from track state to narrow search state. When slice operation is performed, only one target’s range estimation result is obtained within $q_{t}T_{t}$, compared to full-process simulation. This reduces the data rate by a factor of $1/q_{t}$. However, the range information output will be delayed with the slicing approach because range data of the middle CPI is used to represent those of $q_{t}$ CPIs. The latency is $\left\{nT_{t}\left|n=-\left\lceil q_{t}/2\right\rceil ,-\left\lceil q_{t}/2\right\rceil +1,\ldots ,\left\lceil q_{t}/2\right\rceil \right.\right\}$. The range measurement bias of the α-th CPI among $q_{t}$ CPIs can be expressed as $\Delta d_{\alpha }=T_{t}v_{t}\gamma$. The maximum range measurement bias with space slicing method is $q_{t}T_{t}v_{t}/2$. It is obvious that the range measurement error decreases with the reduction in the granularity $\Delta d_{t}$.

In order to make the target parameter error analysis easier to understand, the theoretical error expressions for the full-process and space slicing method are summarized in Table 1. From the analysis in Section 3.3 and Table 1, the range error depends on $q_{t}$ and $T_{t}$. For a constant speed radar, the velocity and angular error are almost unchanged compared with the full-process method.

It is noted that the above error analysis is based on the assumption of the constant radar speed and fixed beam pointing direction within each slice. This is true for the relative constant radar speed scenarios. In the dynamic scenarios, the radar speed varies quickly, which results in the increment of the range error. The velocity and angular error remain unchanged because the variations in a slice are within a resolution bin [42]. In order to decrease the range error, an adaptive strategy will be considered in the future.

## 4. Experimental Results and Analysis

In this section, two examples are used to verify the performance of the proposed method. The simulation parameters are set as follows. The distance between the radar and the target varies from 60 km to 5 km. The initial azimuth and elevation of the target are 0.6350° and 9.8927° in the radar Cartesian coordinate, respectively. The search, acquisition, track, and the narrow search state are included in the entire simulation process. And the broadband blocking jamming is used to interference the search, acquisition, and narrow search state. The R-VGPO jamming is for track state. The signal bandwidth is 5 MHz for the search and narrow search state and 100 MHz for acquisition and track state. The jamming signal bandwidth is set as 50 MHz for broadband blocking jamming and 100 MHz for R-VGPO jamming. The SNR and signal-to-jamming ratio (SJR) vary from −11.51 dB to −4.48 dB and −50.54 dB to −47.02 dB for broadband blocking jamming when the radial distance decreases from 60 km to 40 km, respectively. And the SNR varies from −17.48 dB to 18.63 dB for R-VGPO jamming when the radial distance decreases from 40 km to 5 km, while the SJR is −5 dB for the R-VGPO jamming. The Sinc function is utilized to depict the radar pattern.

### 4.1. Comparison of Simulation Results Between the Full-Process and Space Slicing Simulation

**Figure 10:**
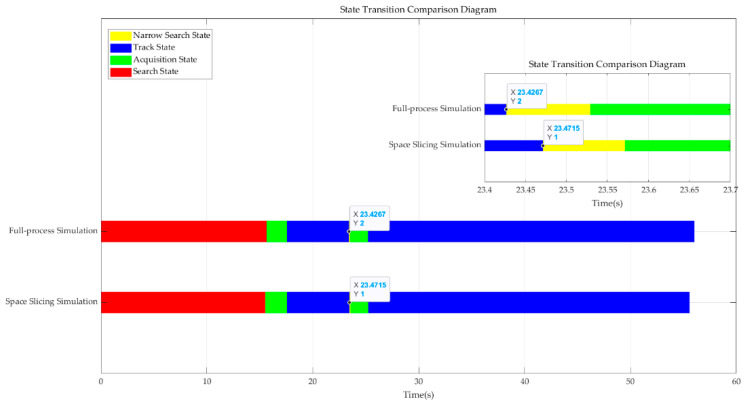
Comparison of radar operating states transition for full-process and space slicing method.

Based on the previously discussed monopulse radar processing workflow, assume the radar undergoes curved motion relative to the target over an operational period of 55 s. We assume that the radar starts to search the target and then determine and track the target under broadband blocking jamming and R-VGPO jamming. The radar loses the target and switches to narrow search state due to the R-VGPO jamming. It reenters the track state after target acquisition. The space slicing granularity for range in the respective radar states is approximately 3 km for the search state, 1.6 km for the acquisition state, 0.15 km for the track state, and 0.2 km for the narrow search state. Additionally, in the track state, slicing occurs approximately every 25 CPIs, dividing the specified spatial domain into slices corresponding to the various radar states. The time–state transition diagram for both full-process and space slicing simulations is illustrated in Figure 10.

As depicted in the diagram, broadband blocking jamming causes multiple rounds of search scans to fail. Upon a successful search, the radar transitions to the acquisition state to confirm the target before entering the stable track state. When the target is being stably tracked, range–velocity pull-off jamming is applied, causing the radar to track the false target instead. Once the jamming ends, the radar loses the false target, resulting in the failure of stable tracking for the real target. The radar then reenters the narrow search state to reacquire and confirm the target, transitioning once again to the stable track state. It is noted that the track state transition to the narrow state with space slicing method is 0.0048 s later than the full-process simulation. This is because the middle CPI is used to represent the 25 CPIs in space slicing method, which coincides with theoretical analysis in Section 3.

**Figure 11:**
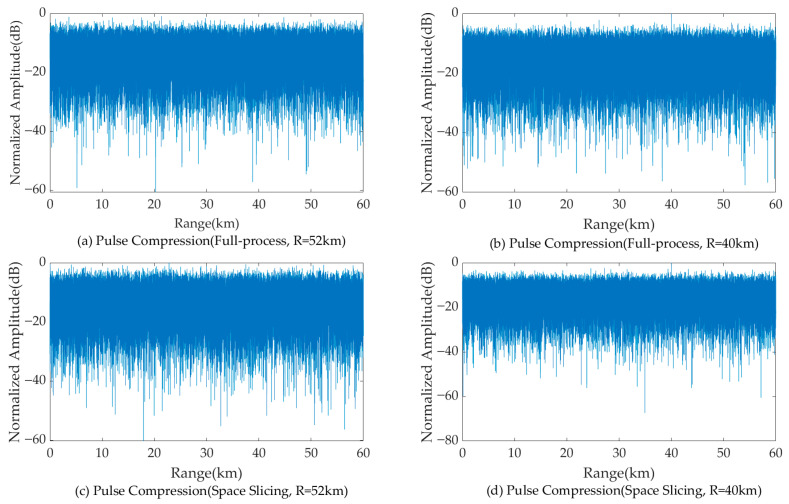
Pulse compression results with broadband blocking jamming.

**Figure 12:**
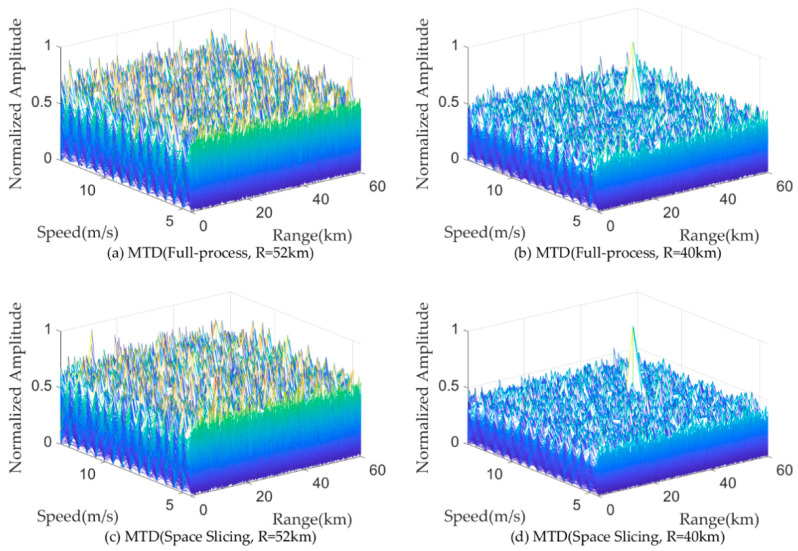
MTD results with broadband blocking jamming.

Figure 11 and Figure 12 illustrate the pulse compression and MTD results for a target located at 50 km and 42 km for the search state in full-process simulation and the space slicing method. The pulse width and PRI are set as 200 us and 600 us for the search state, respectively. There are 16 pulses in a CPI. The SNR are, respectively, −9.09 dB and −4.48 dB for the target located at 50 km and 42 km. The SJR are, respectively, −49.35 dB and −47.02 dB. From the figure, for the two methods, the target located at 50 km cannot be detected since the power of the broadband blocking jamming signal is larger than that of the echo (see Figure 11a,c and Figure 12a,c). On the other hand, when the radial distance decreases, the target can be detected because the power of the echo is larger than that of the jamming (see Figure 11b,d and Figure 12b,d). The comparison of the results reveals that both methods achieve similar target detection outcomes. This is because the echo signal from the same beam position is used to perform the simulation.

**Figure 13:**
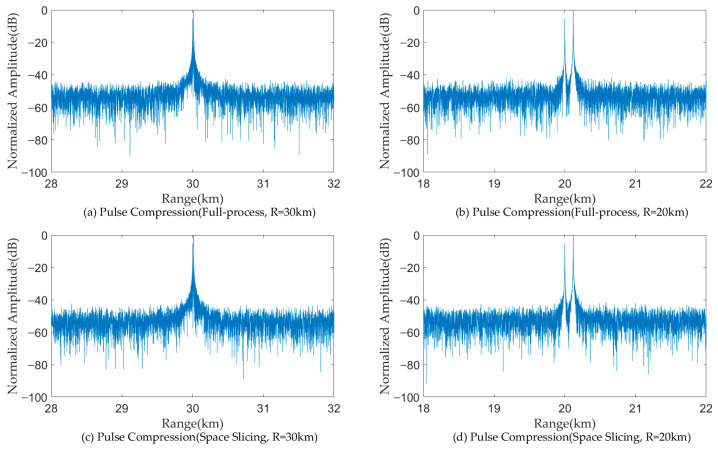
Pulse compression results with R-VGPO jamming.

**Figure 14:**
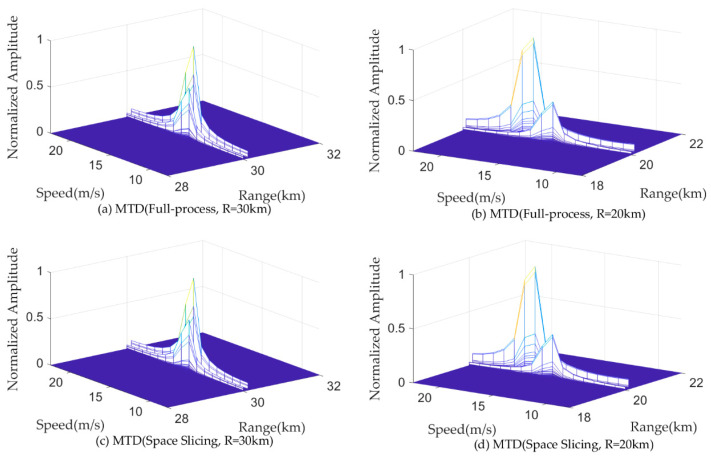
MTD results with R-VGPO jamming.

Figure 13 and Figure 14 illustrate the pulse compression and MTD results for a target located at 30 km and 20 km for the track state in full-process simulation and the space slicing method. The R-VGPO jamming is added to the simulation. The pulse width and PRI are set as 50 us and 250 us for the track state, respectively. The SNR are, respectively, −12.49 dB and −5.44 dB for the target located at 30 km and 20 km. The SJR is set as −5 dB. In Figure 13a,c and Figure 14a,c, the range and velocity of the false target is the same as those of the true target. During this period, the radar can detect and track the false target. The false and true target can be distinguished in Figure 13b,d and Figure 14b,d. Therefore, the radar can detect and track false target due to the larger signal power. The proposed method has the same performance as the full-process method. The reason is the same as that in Figure 11 and Figure 12.

Under computational conditions using an Intel i7-13700H 24 GHz CPU/16G Byte RAM and MATLAB R2023a, the full-process simulation time consumption is approximately 6307.44 s. In contrast, using the pre-generated space slicing database for full-process anti-jamming simulation is only about 0.36 s under the same hardware conditions. Compared to the full-process simulation, the space slicing method neglects a significant portion of signal processing process. Instead, it generates slice data results including target range, velocity, and angles by matching and retrieving data from the slice database based on the provided radar parameters, jamming parameters, strategies, and trajectory information. This approach substantially accelerates computation and improves simulation efficiency.

### 4.2. Simulations of Space Slicing Error

Based on the preceding discussion, the space slicing method proves to be an effective approach for monopulse radar anti-jamming simulation. However, it is not entirely consistent with full-process simulations, as it introduces certain errors. Below, we compare and analyze the errors arising from the space slicing method relative to full-process monopulse radar simulation. Considering the trade-off between the estimation accuracy and computational efficiency, 5 CPI is regarded as a slice in the following simulations. The root-mean-square errors (RMSEs) of the estimated range, velocity, and angular are used to evaluate the parameter estimation performance and are defined as

(23) $$RMSE=\sqrt{\frac{1}{N_{mt}}\sum_{q=1}^{N_{mt}}{\left(\xi_{q}-{\overset{⌢}{\xi }}_{q}\right)}^{2}}$$

where $N_{mt}$ is the number of the Mont Carlo trail, $\xi_{q}$ represents the *q*-th real value of the range, velocity, and angular of the target, and ${\overset{⌢}{\xi }}_{q}$ represents the *q*-th estimation of the range, velocity, and angular of the target.

The RMSE for the space slicing method is defined as:

(24) $$RMSE_{s}=\sqrt{\frac{1}{N_{mt}}\sum_{q=1}^{N_{mt}}{\left(\xi_{q,\alpha }-{\overset{⌢}{\xi }}_{q,\beta }\right)}^{2}},\beta =\left\lfloor \alpha /q_{t}\right\rfloor \cdot q_{t}+\left\lceil q_{t}/2\right\rceil$$

where $\xi_{q,\alpha }$ represents the *q*-th real value of the range, velocity, and angular of the target in *α*-th CPI and ${\overset{⌢}{\xi }}_{q,\beta }$ represents the *q*-th estimation of the range, velocity, and angular of the target in *β*-th CPI, which is regarded as the estimation of the slice.

The two simulations are provided. The R-VGPO jamming is considered in the track state for the first simulation and angular deceptive jamming is for the second one.

#### 4.2.1. Error Analysis for R-VGPO Jamming

**Figure 15:**
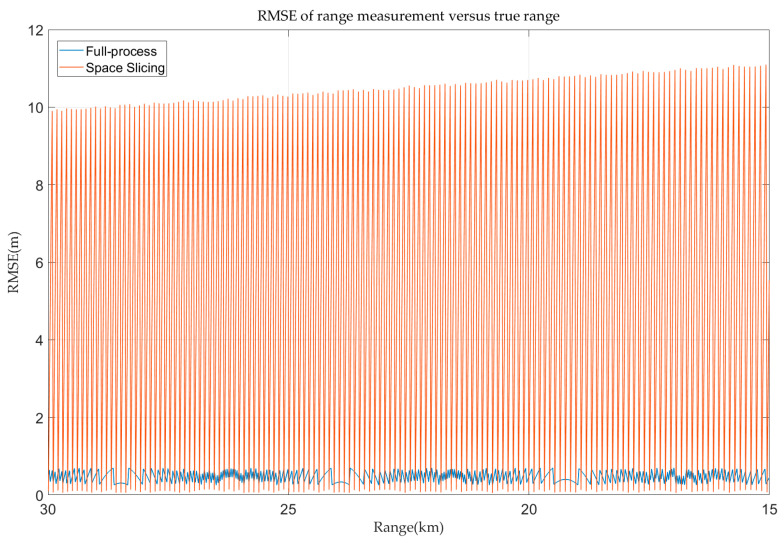
Range error in track state for R-VGPO jamming.

**Figure 16:**
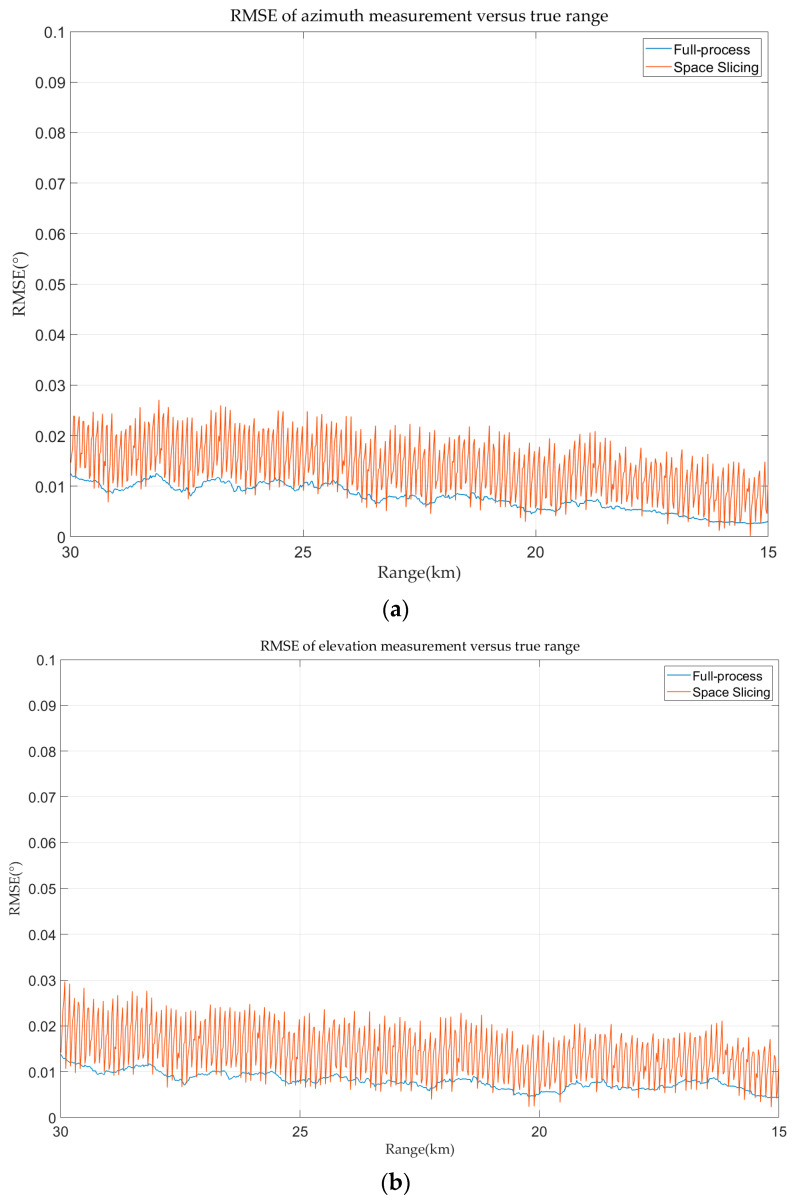
Angular error in track state for R-VGPO jamming. (**a**) Azimuth error in track state for R-VGPO jamming. (**b**) Elevation error in track state for R-VGPO jamming.

In the stable track state, the space slicing method aggregates several CPIs into a single slice in a spatial domain, with the output of that slice approximating the collective results of the CPIs it represents. However, this data compression inherently reduces precision. As shown in Figure 15, the range error of the full-process method is below 0.75 m. This is because the sampling rate in the track state is set as 200 MHz. The range error depends on the sampling rate in the high SNR scenarios. The range error exhibits periodic variations within a range of approximately 0–10 m. The maximum value is consistent with the theoretical expression in Table 1. This periodicity arises because the CPI closest to the slice center incurs the smallest error, while CPIs farther away from the slice center contribute larger errors. Once CPIs exceed the current slice’s boundaries and fall into the adjacent slice, the error trend resets and mirrors the periodic behavior of the preceding slice. Despite these errors, as illustrated in Figure 9, the space slicing method effectively simulates the stable track state. Note that the oscillatory fluctuations in RMSE in Figure 15 and Figure 16 are due to the way our algorithm selects the middle estimate within each slice as its output. However, the true value within each CPI varies, resulting in large spikes in RMSE at regular intervals.

The azimuth and elevation errors for full-process and space slicing are calculated and demonstrated in Figure 16. The azimuth and elevation estimation error decreases with the decrease in the range for the two methods. The reason is that the angular error of the monopulse tracking technique is mainly affected by SNR. The SNR increases with the decrease in the range. The variation in the angular estimation error for the space slicing method behaves the same as that in Figure 15 due to the similar reason. The difference is that the error variation is relatively small since the distance between the center and the boundary of a slice is very small compared with the distance between the radar and target.

**Figure 17:**
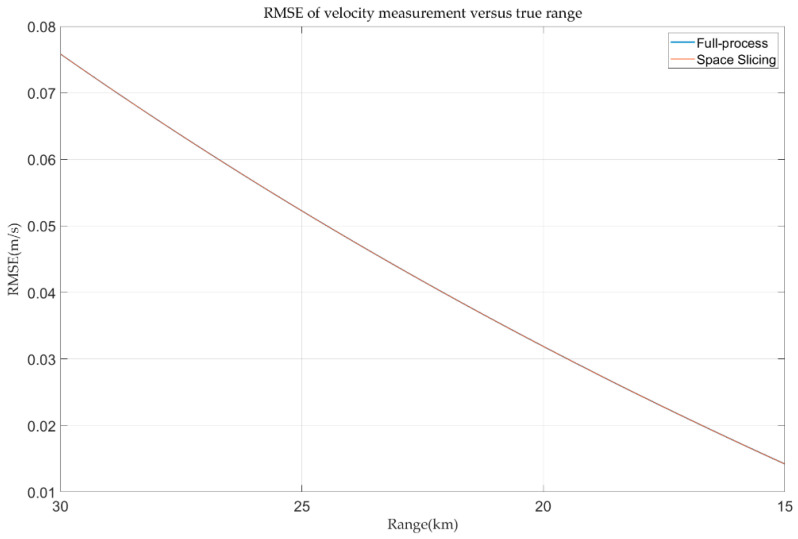
Velocity error in track state for R-VGPO jamming.

Additionally, the velocity measurement errors with full-process and space slicing method in the track state are depicted in Figure 17. The velocity measurement errors of both methods are nearly identical, remaining within 0.07 m/s. This small error is attributed to the minimal velocity variation of the target, which does not exceed a single velocity resolution cell, ensuring that both full-process and space slicing methods produce consistent Doppler channel-based velocity measurements. Therefore, the space slicing method has little effect on the true velocity information of the target in the stable tracking state.

#### 4.2.2. Error Analysis for Angular Deceptive Jamming

**Figure 18:**
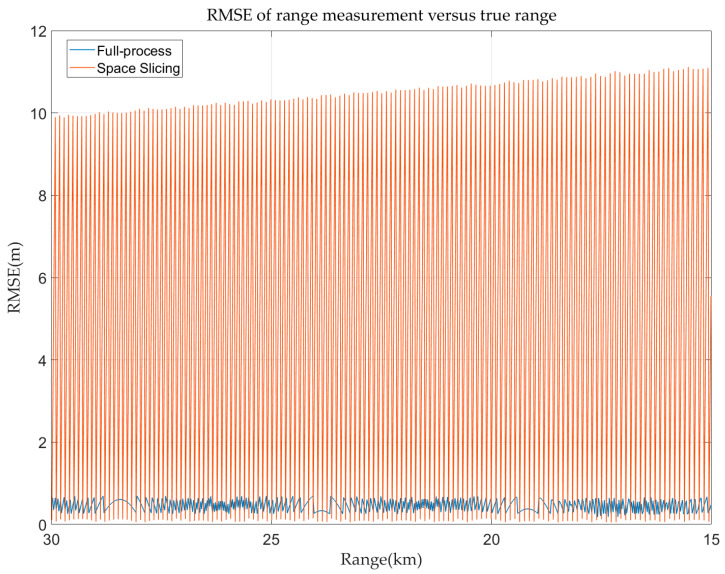
Range error in track state for angular deceptive jamming.

**Figure 19:**
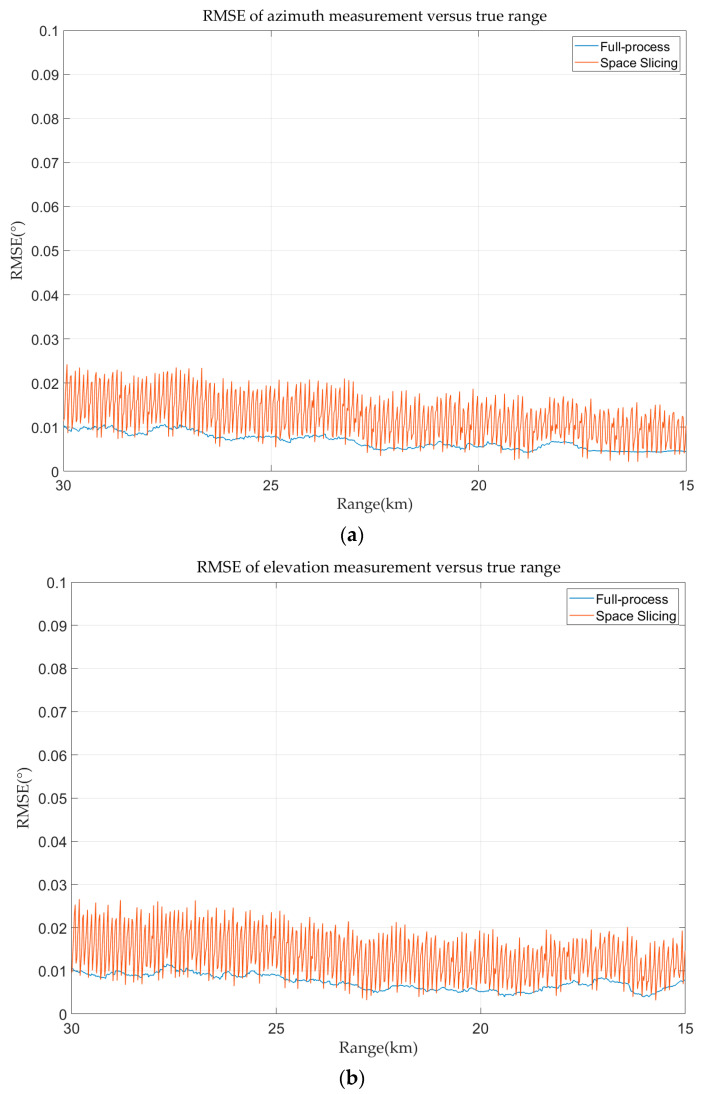
Angular error in track state for angular deceptive jamming. (**a**) Azimuth error in track state for angular deceptive jamming. (**b**) Elevation error in track state for angular deceptive jamming.

**Figure 20:**
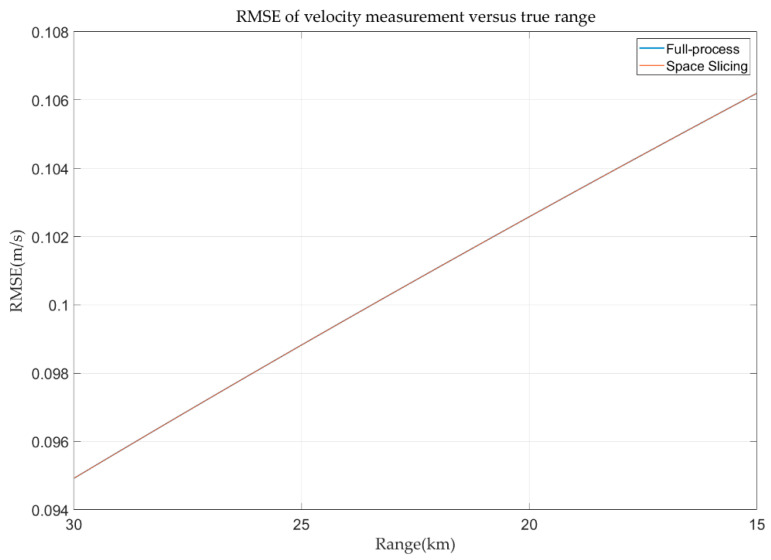
Velocity error in track state for angular deceptive jamming.

The angular deceptive false target is considered in this simulation. At the beginning, the false target and the true target locate at 30 km. And, then, the false target gradually drifts away from the true target at a speed of 9.1918 m/s. The angular deceptive is formed when the false target is detected by radar. The false target disappears at 15 km. During this period, the false target is always in the radar beams. The SJR is set as −3 dB. The range, velocity, and angular error curves of the false target are depicted in Figure 18, Figure 19 and Figure 20. From Figure 18 and Figure 19, the range and angular error behave the same as those in Section 4.2.1. This is because the simulation parameters and motion characteristic are the same in the two simulations. The velocity error in Figure 20 is different to that in Figure 17. The reason is that the velocity of the false target gradually drifts away from the sampling point, while the velocity of the true target is gradually close to the sampling point.

## 5. Conclusions

This study proposed a space-slicing-based simulation algorithm for monopulse radar under jamming conditions that significantly enhances simulation efficiency. The radar signal processing workflow was analyzed, and suppression and deceptive jamming signal models were established. Different space slicing strategies were designed for different radar operating states, and the target parameter estimation precision of the space-slicing-based radar simulation system was assessed for each state. Simulation experiments demonstrate that the proposed method achieves comparable velocity and angle estimation accuracy to full-process simulations, with a distance estimation error of less than 100 m. The time consumption is decreased to 0.36 s with the space slicing method, which improves the simulation efficiency dramatically.

## Figures and Tables

**Table 1:** Comparison of the target parameters theoretical error for full-process and space slicing method.

Parameters	Full-Process Method	Space Slicing Method
range	$\frac{c}{4\pi B\sqrt{2SNR}}$, *B* is the signal bandwidth [41]	$\frac{c}{4\pi B\sqrt{2SNR}}+T_{t}v_{t}\gamma$
velocity	$\frac{\sqrt{6}\lambda }{4\pi \tau \sqrt{SNR}}$τ is the pulse width [41]	$\frac{\sqrt{6}\lambda }{4\pi \tau \sqrt{SNR}}+\Delta v_{\alpha }$$,\;\Delta v_{\alpha }$ see (20)
angular	$\frac{\sqrt{6}\theta_{3dB}}{1.77\pi \sqrt{SNR}}$$,\;\theta_{3dB}$ is 3 dB beam width [41]	$\frac{\sqrt{6}\theta_{3dB}}{1.77\pi \sqrt{SNR}}+\Delta \theta_{\alpha }$$,\;\Delta \theta_{\alpha }$ see (16)

## Data Availability

The original contributions presented in this study are included in the article. Further inquiries can be directed to the corresponding author.

## Abbreviations

The following abbreviations are used in this manuscript:

STK	Systems Tool Kit
PRI	Pulse Repetition Interval
DSP	Digital Signal Processor
FPGA	Field Programmable Gate Array
CPUs	Central Processing Units
GPUs	Graphics Processing Units
R-VGPO	Range–Velocity Gate Pull Off
SNR	Signal-to-Noise Ratio
MTD	Moving Target Detection
RD	Range–Doppler
CFAR	Constant False Alarm Rate
FFT	Fast Fourier Transform
LFM	Linear Frequency-Modulated
LOS	Line of Sight
RF	Radio Frequency
CPI	Coherent Processing Interval
